# Supplementary material for: Ex vivo pretreatment of human vessels with siRNA nanoparticles provides protein silencing in endothelial cells
Source: Nat Commun. 2017 Aug 4;8:191. doi: 10.1038/s41467-017-00297-x (PMC5543113; doi:10.1038/s41467-017-00297-x)
Supplement: Supplementary file 1 — Supplementary Information [file 41467_2017_297_MOESM1_ESM.pdf]

Description of Supplementary Files

File Name: Supplementary Information
Description: Supplementary Figures

File Name: Peer Review File

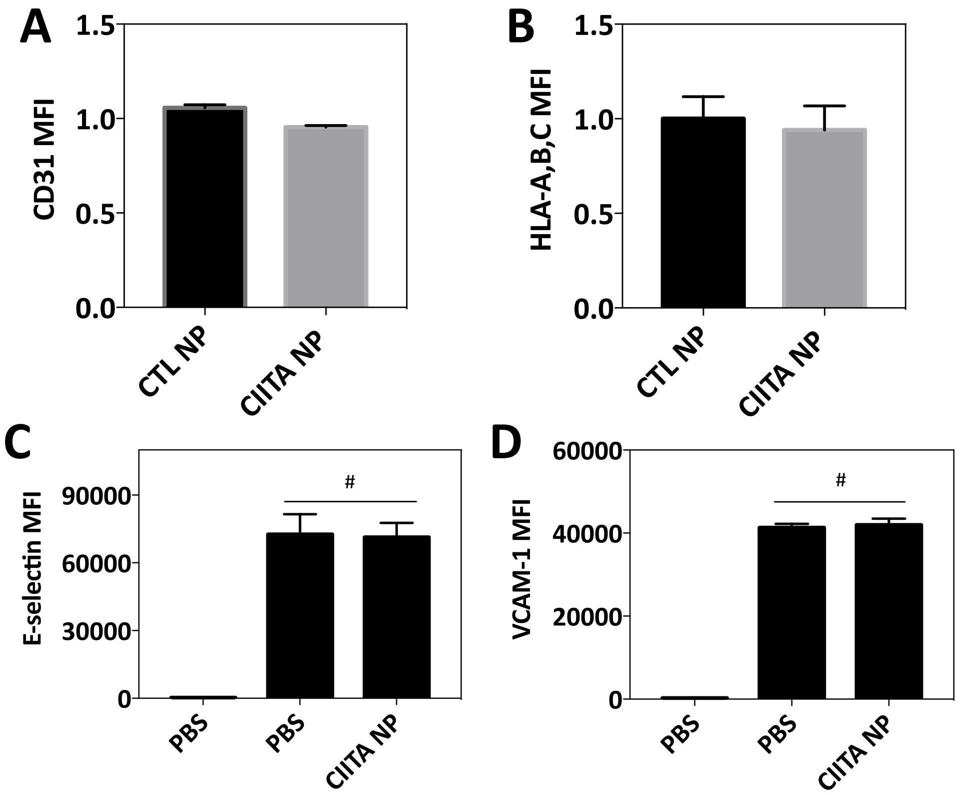

Supplementary Figure 1: NP-mediated knockdown is specific for MHC class II. (A, B) Expression of other EC surface proteins (CD31 and HLA-A,B,C) not targeted by CIITA siRNA was measured using flow cytometry. (C, D) Expression of E-selectin and VCAM-1 was measured using flow cytometry. # denotes addition of TNF-α to induce E-selectin and VCAM-1 expression. Data shown is mean ± s.d. (n = 4).

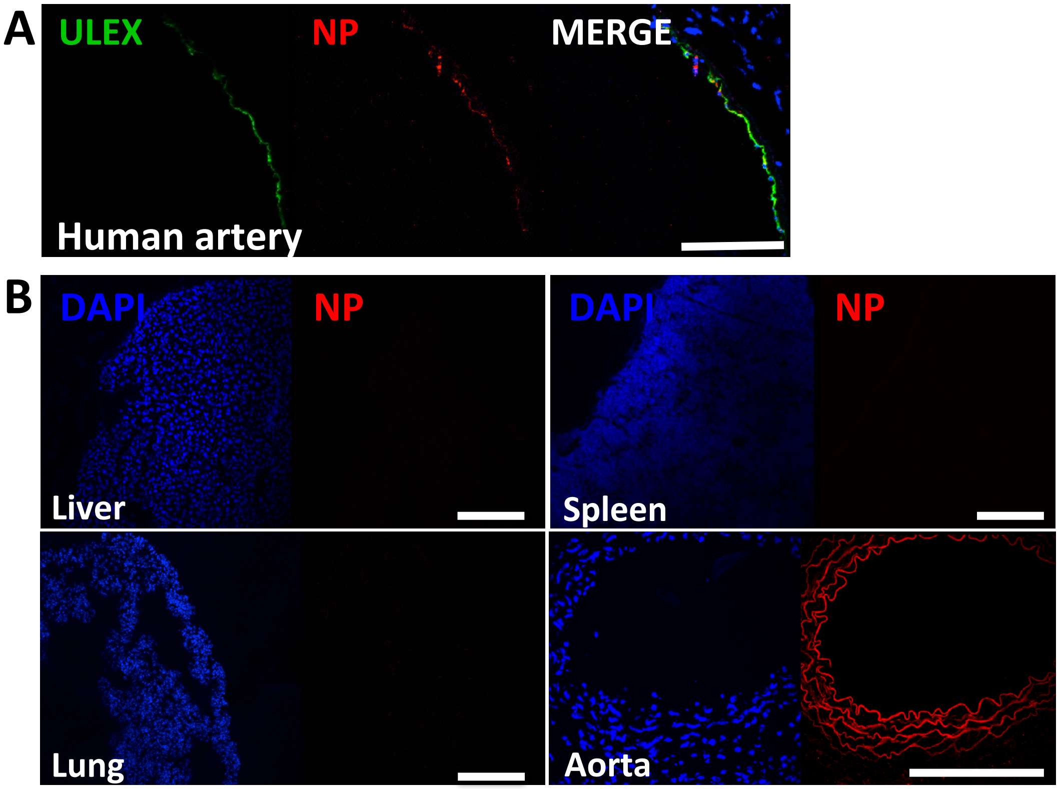

Supplementary Figure 2: NPs are localized to human arterial ECs after transplantation. (A) NP are retained by endothelial cells on the arterial allograft for at least 7 days after transplantation. Human coronary arteries were incubated in culture medium containing 0.2mg mL-1 fluorescent NPs (red) for 6 hours and transplanted into SCID/beige mice. After 7 days, arteries were harvested, sectioned, and stained using anti-Ulex antibody (green) and DAPI (blue), scale bar: 50 μm. (B) Liver, spleen, lungs, and aorta were also harvested after 7 days. Tissues were stained using DAPI and imaged on a confocal microscope, scale bar: 200 μm.

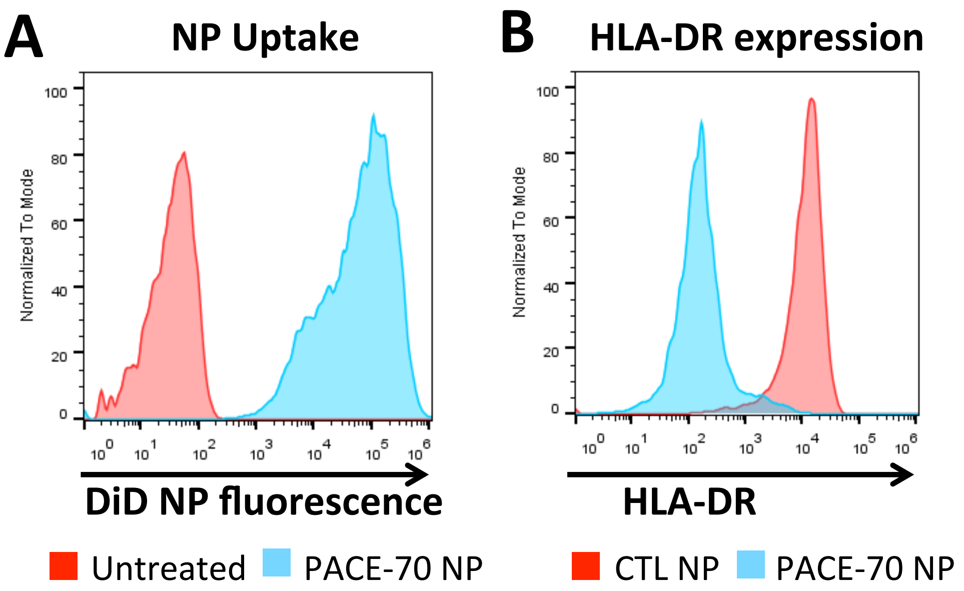

Supplementary Figure 3: Sample graphs of NP uptake and MHC class II knockdown. (A) Representative flow cytometry graph of DiD-loaded PACE-70 NPs uptake by HUVECs. Cells were treated with NPs for 6 hrs. (B) Representative flow cytometry graph of HLA-DR protein knockdown on HUVECs treated with siRNA-loaded PACE-70 NPs. HUVECs were treated with PACE nanoparticles for 6 hrs and then cultured in medium containing hIFN-γ for another 72 hours.
